# Modulation of ovine SBD-1 expression by 17beta-estradiol in ovine oviduct epithelial cells

**DOI:** 10.1186/1746-6148-8-143

**Published:** 2012-08-25

**Authors:** Shiyong Wen, Guifang Cao, Tuya Bao, LanLing Cheng, Haijun Li, Chenguang Du, Yong Tu, Qi Li, Ruizhen Jian, Pengwei Zhao, Wuriliga

**Affiliations:** 1 College of veterinary, Inner Mongolia Agricultural University, Huhhot, 010018, People’s Republic of China; 2 Key Laboratory of Clinical Diagnosis and Treatment Technology in Animal Disease, Ministry of Agriculture, Hohhot, 010018, People’s Republic of China; 3 School of Basic Medical Sciences, Inner Mongolia Medical University, Hohhot, 010059, People’s Republic of China; 4 Vocational and Technical College, Inner Mongolia Agricultural University, Baotou, 014109, People’s Republic of China; 5 Keshiketeng Banner Animal Disease Control Center, Chifeng, 025350, People’s Republic of China; 6 Pathogenic Organisms and Immunology Lab, basic medical college, Inner Mongolia Medical University, Hohhot, 010059, People’s Republic of China; 7 College of animal science, Inner Mongolia Agricultural University, Huhhot, 010018, People’s Republic of China

**Keywords:** Sheep, Oviduct epithelial, SBD-1, Modulation, Signaling pathway

## Abstract

**Background:**

Mucosal epithelia, including those of the oviduct, secrete antimicrobial innate immune molecules (AIIMS). These have bactericidal/bacteriostatic functions against a variety of pathogens. Among the AIIMs, sheep β-defensin-1 (SBD-1) is one of the most potent. Even though the SBD-1 is an important AIIM and it is regulated closely by estrogenic hormone, the regulation mechanism of 17β-estradiol has not been clearly established. We investigated the effects of E_2_ and agonist or inhibitor on ovine oviduct epithelial cells in regard to SBD-1 expression using reverse transcription quantitative PCR (RT-qPCR). In addition, three different pathways were inhibited separately or simultaneously to confirm the effect of different inhibitors in the regulation mechanism.

**Results:**

17beta-estradiol (E_2_) induced release of SBD-1 in ovine oviduct epithelial cells. SBD-1 expression was mediated through G-protein-coupled receptor 30 (GPR30) and Estrogen Receptors (ERs) activation in ovine oviduct epithelial cell. Inhibition of gene expression of protein kinase A (PKA), protein kinase C (PKC), and nuclear factor kappa-light-chain-enhancer of activated B cells (NF-κB) led to a decreased SBD-1 expression.

**Conclusions:**

Taken together, E_2_-induced up-regulation of SBD-1 expressions were GPR30-dependent during prophase and ERs-dependent during later-stage in ovine oviduct epithelial cells, and we assume that the effect was completed by the PKA, PKC, and NF-κB pathways simultaneous.

## Background

Ovine oviduct epithelial cells are effective barriers for microorganisms and actively participate in the initiation of innate host defense. Unfortunately, salpingitis is the one of most common and serious infections in reproductive system diseases [1]. However, there has been a dramatic worldwide increase in antibiotic resistance in pathogens in the past several decades. Thus, there is an urgent need to develop new and innovative, non-antibiotic approaches to prevent and manage this disease [2,3]. Defensins are antimicrobial peptides of innate immunity functioning by non-specific binding to anionic phospholipids in bacterial membranes.

β-defensins are produced directly by epithelial cells, and combat infection both through direct microbicidal action and by modulation of cell-mediated immunity [4-7]. These peptides are involved in the innate immunity mechanisms and act directly against bacteria, viruses, and fungi, due to their bactericidal and cytotoxic activity [8]. Moreover, they have been suggested as effector molecules in host defense, interacting with many target cells and tissues [9]. So far, only two β-defensin genes have been identified in sheep: β-defensin 1 (SBD-1) and β-defensin 2 (SBD-2), although genomic studies suggest more have yet to be discovered [10]. Recent studies have revealed that the SBD-1 is an important component of the innate immune response of the ovine oviduct.

Nonetheless, there is a paucity of information about the role and regulation of the expression of the oviduct SBD-1 gene in ovine and, in particular, whether E_2_ modulate its expression. In the present study, the expression of mRNA SBD-1 was studied by a PCR (RT-qPCR) assay in oviduct epithelial cells treated with E_2_. Since previous studies have demonstrated that E_2_ provides a stimulus that may regulate defensins, this study investigated the effect of three pathways, PKA, PKC and NF-κB, in SBD-1 expression by oviduct epithelial cultured cells.

## Results

### E_2_ up-regulates SBD-1 expression in ovine oviduct epithelial cells

**Figure 1:**
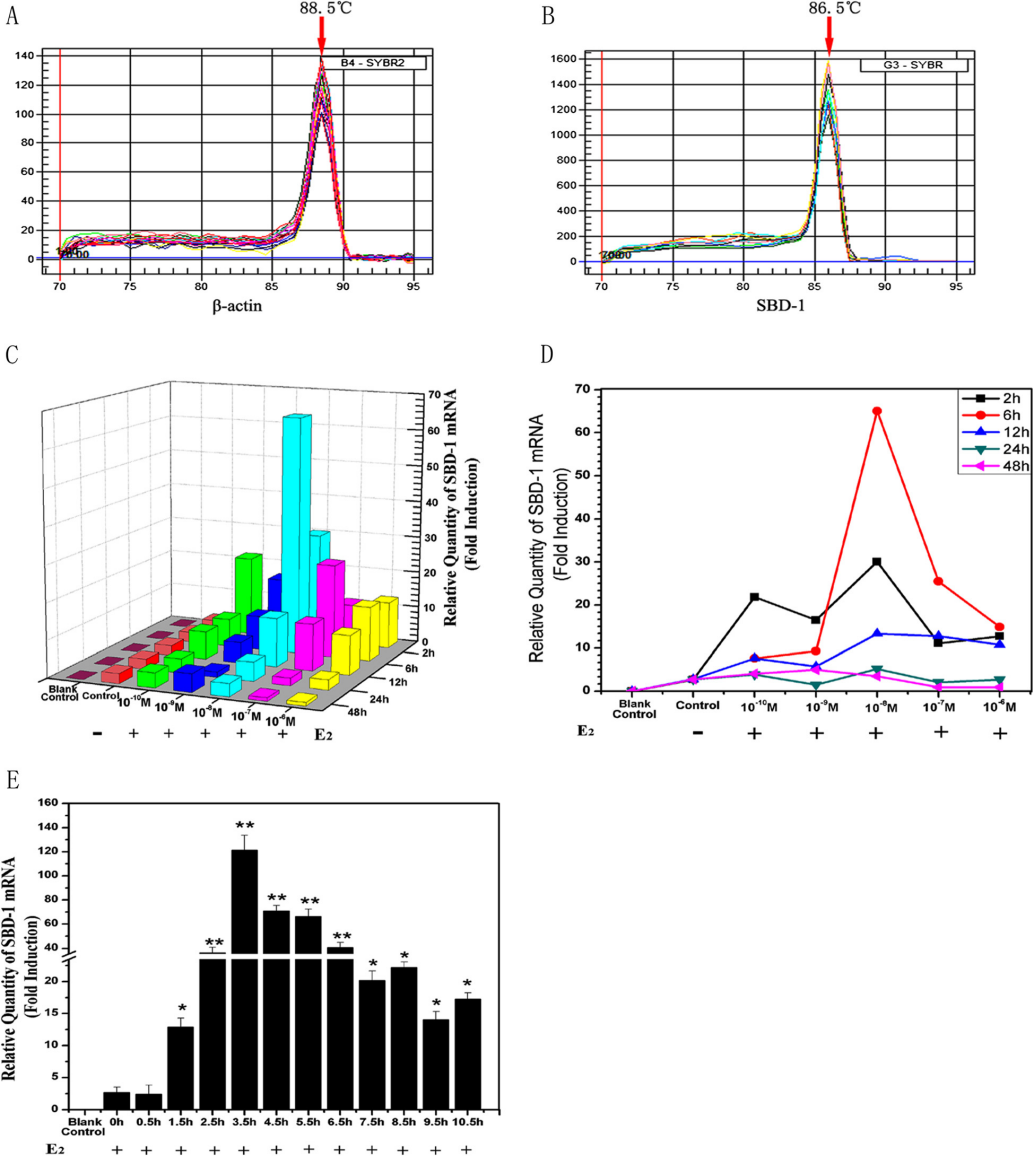
**E**_**2 **_**up-regulates SBD-1 gene expression in ovine oviduct epithelial cells. **Through QPCR, these primers are valid because of the single peak in every dissociation curve using SYBR® Green I. (**A**, **B**) Different concentrations of E_2_ treatment of ovine oviduct epithelial cells increased the mRNA levels of SBD-1. The z-axis or y-axis represents the signal derived from quantitative PCR on total RNA extracted from the cells. (**C**) The x-axis shows the time during which the cells were exposed to 10^-8^ M E_2_. Furthermore, a blank control (water substituted for cDNA) was included in each qPCR reaction mixture, and a control was set up containing water instead of E_2_ in every group. (**E**) The greatest induction was observed in the 10^-8^ M E_2_ treated group at 3.5 h. Different concentrations of E_2_ were added to the various groups, and total RNA extracted at the indicated time points from the ovine oviduct epithelial cells (n = 3). Values are given as mean ± standard deviation. (*: p < 0.05 vs control, **: p < 0.01 vs control).

To find out whether E_2_ can increase expression of SBD-1 mRNA in different concentrations and times, we treated ovine oviduct epithelial cells with different concentrations E_2_ and different times. Figure 1C&D shows the kinetics of SBD-1 expression. As is evident from the figure, the induction of SBD-1 mRNA expression was very rapid after the cells had been treated for 2 h and the greatest induction was observed at 6 h, which suggests that the highest expression levels were around 6 h. Afterwards, although the up-regulation was continuing, the enhanced amplitude gradually decreased, and the lowest induction was at 48 h (Figure 1C). Besides, the concentration of 10^-8^ M provided the greatest induction at every time point except at 48 h, which suggests that the optimum concentration of E_2_ for the cells is 10^-8^ M (Figure 1D). Therefore, we treated the cells with E_2_ (10^-8^ M) to seek the time point of the greatest induction of SBD-1 expression. Figure 1E shows that the greatest induction of SBD-1 expression was observed at 3.5 h and the induction of SBD-1 expression has significant differences from 1.5 to 10.5 h. The change of SBD-1 expression level was especially prevalent between 2.5 to 6.5 h.

### SBD-1 expression was mediated through GPR30 and ERs activation in ovine oviduct epithelial cells

**Figure 2:**
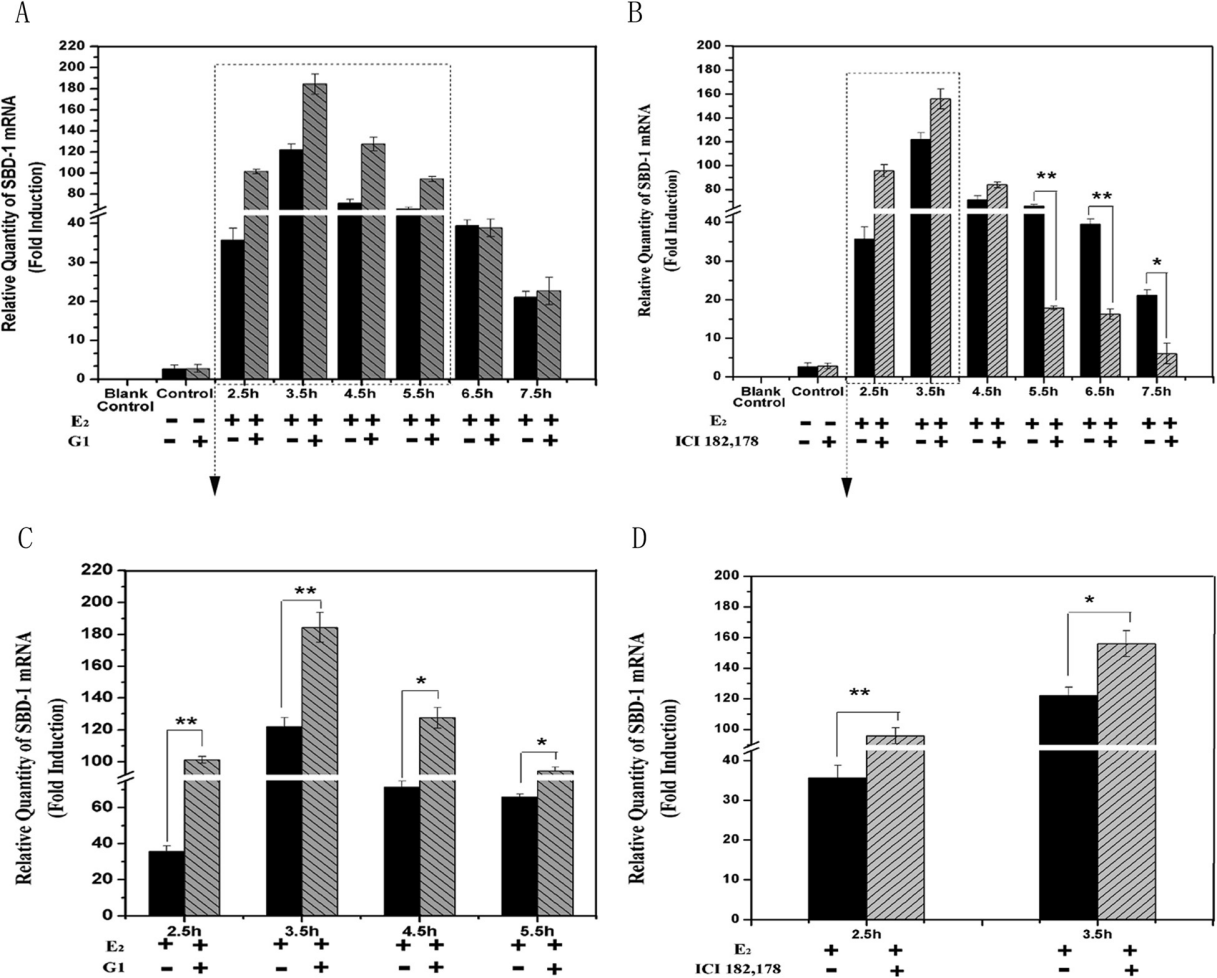
**E**_**2**_**-induced SBD-1 expression in ovine oviduct epithelial cells requires GPR30 and ERs. **(**A**) The cells were treated with the GPR30 agonist G1 (10^-7^ M) and E_2_ (10^-8^ M). (**B**) Other groups of cells were incubated with the inhibitor ICI 182,780 (Anti-ER 10^-7^ M, 1 h) and then added with E_2_ (10^-8^ M). Furthermore, a blank control (water substituted for cDNA) was included in each qPCR reaction mixture, and a control was set up containing water instead of E_2_ or/and G1/ ICI 182,178 in every group. (**C**, **D**) In the magnified inset, there were statistically significant changes between the two different treatments at different times from A or B. Total RNA extracted at the indicated times from the ovine oviduct epithelial cells (n = 3). Values are given as mean ± standard deviation. (*: p < 0.05 vs control, **: p < 0.01 vs control).

Recent studies showed that activation of GPR30 and ERs controls estrogen-responsiveness [11,12]. Therefore, to further investigate a possible role of GPR30 and ERs activation for E_2_-dependent SBD-1 expression, we pre-incubated ovine oviduct epithelial cells with the high-affinity agonist G1 for GPR30 and the inhibitor ICI 182,780 to prevent ERs bind with E_2_. As shown in Figure 2A&C, treatment of cells with E_2_ and G1 resulted in SBD-1 expression increasing from 2.5 to 5.5 h and the effect is not obvious from 6.5 to 7.5 h. Inhibiting ERs activation inhibited the SBD-1 expression (Figure 2B) in E_2_-treated cells from 5.5 to 7.5 h, whereas SBD-1 expression increased from 2.5 to 3.5 h (Figure 2D). Our data demonstrated that activation of GPR30 and ERs by E_2_ was important for SBD-1 expression in ovine oviduct epithelial cells.

### The PKA, PKC and NF-κBpathways were essential in E_2_-induced up-regulation of SBD-1 expression

**Figure 3:**
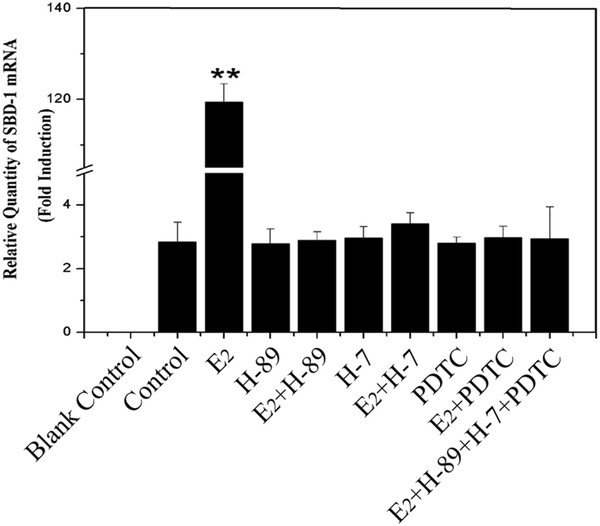
**E**_**2**_**-induced SBD-1 expression in ovine oviduct epithelial cells via cAMP/PKA pathway, PKC pathway and NF-κB pathway. **Cells treated with the PKA (H-89, 50 μM), the PKC (H-7, 50 μM), and NF-κB inhibitors (PDTC, 50 μM) showed significantly lower SBD-1 expression compared to the control. Furthermore, a blank control (water substituted for cDNA) was included in each qPCR reaction mixture, and a control was set up containing water instead of E_2_ or/and inhibitor(s) in every group. Total RNA extracted at the indicated time points from the ovine oviduct epithelial cells (n = 3). Values are given as mean ± standard deviation. (*: p < 0.05 vs control, **: p < 0.01 vs control).

Having demonstrated that the greatest induction was observed at 3.5 h in E_2_-induced up-regulation of SBD-1 expression, we went on to elucidate the pathway by which signal was transduced to the nucleus. Based on the putative mechanisms of estrogen action [13],we tested the hypothesis that the PKA, PKC and NF-κB pathways might be essential in E_2_-induced up-regulation of SBD-1 expression using PKA inhibitor H-89, PKC inhibitor H-7 and NF-κB inhibitor PDTC. Figure 3 shows that the SBD-1 expression was significantly reduced when treated with inhibitor or inhibitors with no difference compared to the control group.

## Discussion

Antimicrobial innate immune molecules produced by the epithelial cells provide the host with a constitutive or immediately inducible defense mechanism against invading pathogens [14]. Meanwhile, non-inflamed tubal and middle ear mucosa have been shown to contain relatively few immunocytes [15,16]. Furthermore, because of the under-developed and premature adaptive immune system in young animals, the role of AIIMs in protecting against pathogens becomes important. This suggests that AIIMs protect the body during the neonatal and early postnatal periods when the adaptive immunity is not yet fully developed.

Among the AIIMs, defensins and cathelicidins are the dominant antimicrobial peptides found in neutrophils and epithelia as components of the early host defenses of mammals against infection, and these peptides have potent microbicidal activity against prokaryotic and eukaryotic pathogens as well as viruses [17]. The expression of defensin in epithelial cells is mediated by certain hormones and is closely related to estrogenic hormone and progesterone [18]. This suggests that E_2_ might be important in the modulation of ovine defensin expression in ovine oviduct epithelial cells. However, there is little information on how the E_2_ regulates the expression of defensins in ovine oviduct epithelial cells by E_2_.

We demonstrate that the gene of SBD-1 was expressed in ovine oviduct epithelial cells through the RT-PCR analysis and sequencing. In addition, using qPCR, there was a single peak in every dissociation curve (Figure 1A&B), and the amplification efficiencies of SBD-1 and β-actin were 0.996 and 0.990. Meanwhile, both slopes were −3.3. Based on this we also demonstrated that E_2_ could up-regulate the expression of SBD-1, especially when treated with 10^-8^ M E_2_ (Figure 1C&D). The mechanism by which E_2_ induces or activates the expression of SBD-1 is not precisely understood, but our data showed that the expression of SBD-1 still occurs in ovine oviduct epithelial cells left untreated. Therefore, E_2_ may be not necessary for the expression of SBD-1 but nay rather play a role as a promoting factor. Moreover, after stimulation with E_2_ at 10^-8^ M, the effect continued for at least 24 h (Figure 1C&D) and peaked at 3.5 h, while, the highest expression level had been sustained for 4 h from 2.5 to 6.5 h (Figure 1E). This data suggests that the expression was activated and reached it’s highest level simultaneously, but only for a short time, after which time it continued as a reduced-level, which was still much higher compared with the non-treated control. This shows that there might be a fast pathway, which could be activated quickly, in the expression of SBD-1 by E_2_.

Previous studies have suggested that the putative mechanisms of estrogen action have two mechanisms: a Non Classical Genomic Mechanism and a Classical Genomic Mechanism. Meanwhile, GPR30 and ERs might play important roles in the two mechanisms [13]. Based on these results, we investigated the role and identity of GPR30 and ERs in the modulation of SBD-1 expression by E_2_ in ovine oviduct epithelial cells. We noted that the expression increased after addition of E_2_ and G1 from 2.5 to 5.5 h and with no change in the E_2_ only treatment from 6.5 to 7.5 h (Figure 2A&C). Meanwhile, after addition of E_2_ and ICI 182,780, the expression diminished quickly from 5.5 to 7.5 h (Figure 2B), however, the expression increased from 2.5 to 3.5 h (Figure 2D). This is consistent with the fact that ICI 182,780 is also an agonist at the membrane estrogen receptor GPR30 [19]. These results suggest that E_2_-induced up-regulation of SBD-1 mRNA expression was dependent on GPR30 and ERs in ovine oviduct epithelial cells at different times. GPR30 plays an important role in Non Classical Genomic Mechanism, activating rapid pathways, while ERs acts with a Classical Genomic Mechanism controlling gene transcription.

In the Non Classical Genomic Mechanism, the cAMP/PKA pathways [20], PKC pathway [21] and NF-κB pathway [22,23] play important roles in the putative mechanisms of estrogen action [13]. Our data showed that there was almost no difference between the untreated and treated cells, which were added with the inhibitors of H-89, H-7, and PDTC either individually or in combination for 3.5 h (Figure 3). These results suggest that the three pathways were indispensable for the expression of SBD-1 by E_2_ in ovine oviduct epithelial cells. Furthermore, we also conclude that the three pathways should be activated simultaneously, or otherwise there would be no effect on the E_2_-induced up-regulation of SBD-1 expression.

## Conclusion

In conclusion, we found that in ovine oviduct epithelial cells E_2_-induced up-regulation of SBD-1 expression was GPR30-dependent during prophase and ERs-dependent during later-stage. Expression of SBD-1 included activation of the cAMP/PKA pathway, PKC pathway and NF-κB pathway via the Non Classical Genomic Mechanism, whereas the precondition that all three pathways were activated simultaneously was essential for this process. Since control of the immune response is crucial to prevent excessive tissue damage and to assure bacterial clearance in salpingitis, the mechanism described above could be important for the host defense in the ovine oviduct.

## Methods

### Animals

This study involved five ewes of the Mongolia breed, 13–15 months old. All sheep were free of parasitic and infectious diseases. The animals were purchased from Tecon Group located in the Urumqi (Xinjiang Autonomous Region, PR China). All animals were cared for by trained animal keepers and fed with hay and a commercial pelleted ration. Water and mineralized salt were available ad libitum. All experimental animals in this study were approved by the Institutional Animal Care and Use Committee (IACUC) at Inner Mongolia Agriculture University.

Animal handling, euthanasia and experimental procedures were carried out in compliance with the Tecon Group regulations (Licence NO. SYXK, Xin, 2010–0005), with the approval of the Animal Ethics Committee of the Inner Mongolia Agriculture University of Inner Mongolia. Euthanasia was done by intravenous injection of barbiturate overdose followed by exsanguination and immediate removal of the oviducts.

### Materials

The ERs inhibitor (ICI 182,780, Cat.No. 1047) was purchased from Tocris (Cambridge, UK), the PKA inhibitor (H-89, Cat.No. B1427), the PKC inhibitor (H-7, Cat.No. I7016), the NF-κB inhibitor (PDTC, Cat.No. P8765) were purchased from Sigma Chem. Co. (Munich, Germany), the GPR30 agonist (G1, Cat.No. 371705) was purchased from Merck (Darmstadt, Germany). All other chemicals used were of analytical grade and obtained from commercial sources.

### Cell culture

Ovine oviduct epithelial cells were isolated based on the protocol by R. Ian Freshney and Mary G. Freshney [24], and all the procedures were performed under sterile conditions. Briefly, ovine oviducts were removed immediately after slaughter and placed in cold PBS supplemented with 1% penicillin/streptomycin (Sigma). Once in the laboratory, oviduct tissue, which was bisected longitudinally by ophthalmology shear, was placed in HBSS (Sigma) with 0.05% pancreatin (Sigma) and 0.02% EDTA (Sigma) and incubated at 37°C for 12 min. After that the inner surface of the oviducts were lightly scraped with a scalpel in Hanks balanced salt solution. Tissue was removed and scraped material (the cells) was agitated by pipetting up and down using a Movette pipette; cells were centrifuged at 800 × g for 3 min and resuspended in HBSS. Repetitions of this step were performed until the HBSS remained almost clear. (This usually takes about 3 repeats of the washing process.) Cells were placed into 25 cm^2^ cell culture flasks (Corning Inc., Corning, NY) with phenol red-free DMEM/F-12 (Thermo) plus 15% (vol/vol) heat-inactivated FCS, 100 μg/ml streptomycin and 100 U/ml penicillin. Cells had been grown for 48 to 72 h until 80 – 90% and passaged into six-well cell culture plates in triplicate. For studying the effect of E_2_, the cells were washed with serum-free DMEM/F-12 and placed under serum-free conditions for 12 h, and were pretreated for one hour with or without chemical agonist of GPR30 (G1) and chemical inhibitors including ICI 182,780, H-89, H-7, PDTC. All cells were maintained in a humidified atmosphere of 5% CO_2_ and 95% air.

### RNA and cDNA preparation

Total RNA from cells was extracted using RNAiso Plus (TaKaRa, Japan) according to manufacturer’s protocol. 1 ml of RNAiso Plus was used for each 10 cm^2^-well of cells and lysed in an RNase free environment. Chloroform was then added (200 μl for each lysate) and the samples were centrifuged at high speed for 15 minutes at 4°C. The aqueous layer was then transferred into a new Eppendorf tube and RNA was precipitated with iso-propanol followed by one wash using 70% ethanol. The RNA precipitate was then dissolved in 15–20 μl of RNase-free water. Digestion of genomic DNA was performed using DNase I (Sigma). The integrity of the extracted RNA was examined by 1% agarose gel electrophoresis; the quantity and purity were assessed by measuring absorbance using a BioPhotometer plus (Eppendorf, Germany). After that, only samples with an OD 260/230 ratio greater than 1.6 and an OD 260/280 ratio between 1.8 and 2 were included in subsequent studies. The cDNA was synthesized in a volume of 20 μl using 1 μg of total RNA from each sample and PrimeScript^TM^ RT reagent Kit (TaKaRa, Japan) in time, and all cDNA was stored at −20°C.

### Efficiency Measurements and Real-Time qPCR

Amplification efficiency (Eff = 10^(−1/slope)-1)^ values were determined in parallel runs with cDNA samples for each gene during RT-qPCR. Preliminary qPCR assays, using the Cq slope method described below, were used to measure the two genes’ amplification efficiency. This method involves generating a dilution series of the target template and determining the Cq value for each dilution. A plot of Cq versus log cDNA concentration was constructed. All primers (Table 1), which were designed and synthesized from Sangon Biotech (Shanghai, China), spanned the exon borders of the gene to avoid amplification of any contaminating genomic DNA.

**Table 1:** The primers

**Gene (Accession No.)**	**Primers (5′-3′)**	**Product size**
SBD-1(U75250)		
**F**	GGCTCCATCACCTGCTCCTC	206 bp^a^
**R**	CGTCTTCGCCTTCTGTTACTTCTT	
β-actin(U39357)		
**F**	GTCACCAACTGGGACGACA	208 bp
**R**	AGGCGTACAGGGACAGCA	

^a^ bp, base pairs.

### Quantification of SBD-1 and reference gene transcript copy number for the cDNA

Samples were determined by RT-qPCR analysis carried out on the iCycler iQ5™ Real-Time PCR Detection System (Bio-Rad, Hercules, CA, USA) using gene specific primers, according to the manufacturer’s instructions. Each 20 μl reaction was run in triplicate and consisted of: SYBR® Premix Ex Taq^TM^ II (2×, Perfect Real Time, TakaRa, Japan) 10.0 μl, 0.4 μl (10 μM) each of the forward and reverse primers, cDNA template 2.0 μl, and ddH_2_O 7.2 μl used to normalize the fluorescent reporter signal between reactions. RT-qPCR conditions consisted of 1 cycle at 95°C (30 seconds); 50 cycles of 95°C (20 seconds), 63°C (30 seconds), 72°C (20 seconds), 82°C (10 seconds); 1 cycle at 72°C (7 minutes); followed by a 51-step melting-curve analysis (initial temperature 70°C, increasing 0.5°C every 6 seconds), including a negative control consisting of PCR-grade water. Data analysis was performed using the Bio-Rad iQ5 Software 2.1 and the results were expressed as fold-change in relative mRNA expression level, calculated using the ΔΔCt method with β-actin as the reference gene and the non-treated cells as baseline. Dissociation curves and 1% agarose gel electrophoresis, containing ethidium bromide were visualized under ultraviolet light and used to verify the presence of single PCR products. After that, the amplicons were cloned into pMD19-T vector (TaKaRa, Japan) and confirmed by sequencing (Shanghai, China).

### Statistical Analysis

Data were analyzed using the SPSS Statistics 17.0 (SPSS Inc., Chicago, IL, USA). Results are expressed as mean ± SD of at least three independent experiments. Differences between control and samples were compared by matched pair t-test and were considered significant when the P value was ≤ 0.05.

## Abbreviations

AIIMs: antimicrobial innate immune molecules; PBS: phosphate-buffered saline; HBSS: Hanks balanced salt solution; EDTA: ethylenediaminetetraacetic acid; DMEM/F12: Dulbecco’s Modified Eagle Medium: Nutrient Mixture F-12; FCS: fetal calf serum; Cq: Quantification cycle.

## Competing interests

The authors declare that they have no competing interests.

## Authors’ contributions

SW carried out experimental design, RNA and cDNA preparation, real-time qPCR experiments, statistical analysis, manuscript and figures preparation, GC and HL carried out research design, experimental design, TB and RJ carried out experimental design, RT-qPCR experiments, figure preparations, LC and QL carried out cell culture, RNA and cDNA preparation, YT and PZ carried out experimental design, W and CD carried out statistical analysis. All authors read and approved the final manuscript.
